# Long Noncoding RNA Expression Profiles of Periodontal Ligament Stem Cells from the Periodontitis Microenvironment in Response to Static Mechanical Strain

**DOI:** 10.1155/2021/6655526

**Published:** 2021-04-10

**Authors:** Jia Liu, Yan Zhao, Qiannan Niu, Ni Qiu, Shuangyun Liu, Chunrong Li, Cuixia Li, Pei Miao, Libo Yan, Qiang Li, Zuolin Jin

**Affiliations:** ^1^State Key Laboratory of Military Stomatology and National Clinical Research Center for Oral Diseases and Shaanxi Clinical Research Center for Oral Diseases, Department of Orthodontics, School of Stomatology, The Fourth Military Medical University, Xi'an, Shaanxi 710032, China; ^2^Department of Stomatology, 920 Hospital of PLA, Kunming, Yunnan 650032, China; ^3^Department of Stomatology, 260 Hospital of PLA, Shijiazhuang, Hebei 050041, China; ^4^State Key Laboratory of Military Stomatology & National Clinical Research Center for Oral Diseases & Shaanxi International Joint Research Center for Oral Diseases, Department of General Dentistry & Emergency, China

## Abstract

During the period of orthodontic tooth movement, periodontal ligament stem cells (PDLSCs) play an important role in transducing mechanical stimulation and tissue remodeling. However, our previous studies verified that the periodontitis microenvironment causes damage to the biological functions of PDLSCs and abnormal mechanical sensitivity. Long noncoding RNAs (lncRNAs) participate in the inflammatory pathogenesis and development of many diseases. Whether lncRNAs are abnormally expressed in PDLSCs obtained from periodontal tissues of periodontitis patients (PPDLSCs) and whether putative lncRNAs participate in the mechanotransductive process in PDLSCs remain poorly understood. First, we subjected PDLSCs obtained from healthy periodontal tissues (HPDLSCs) and PPDLSCs to static mechanical strain (SMS) with 12% elongation at 0.1 Hz frequency using an FX-4000T system and screened overall lncRNA profiles in both cell types by microarray. Among lncRNAs with a fold change (FC) > 20.0, 27 lncRNAs were upregulated in strained HPDLSCs, and 16 lncRNAs (9 upregulated and 7 downregulated) were detected in strained PPDLSCs. For mRNAs with FC > 20.0, we detected 25 upregulated mRNAs and one downregulated mRNA in strained HPDLSCs and 7 upregulated and 5 downregulated mRNAs in strained PPDLSCs. Further enrichment analysis showed that, unlike HPDLSCs with annotations principally involving transduction-associated signaling pathways, dysregulated mRNAs in PPDLSCs are mainly responsible for pathological conditions. Moreover, coexpressed lncRNA-mRNA networks confirmed the pathological state and exacerbated inflammatory conditions in strained PPDLSCs. Taken together, when compared with strained HPDLSCs, various lncRNAs and mRNAs were dysregulated in PPDLSCs under mechanical forces, implicating the response of lncRNAs in PPDLSCs to mechanical stress. Moreover, we provide potential lncRNA targets, which may contribute to future intervention strategies for orthodontic treatment in periodontitis patients.

## 1. Introduction

Periodontitis is a chronic inflammatory disease that causes irreversible periodontal attachment damage [1]. During the pathological process, osteoblasts are distinctly suppressed, whereas osteoclastogenesis becomes hyperactive [2]. Because of the typically high morbidity rate and obvious clinical manifestation of tooth extrusion, space, and labial drifting, many adult patients with periodontitis seek orthodontic treatment to achieve both esthetic restoration and functional restoration [3]. Periodontal ligament stem cells (PDLSCs) are considered an attractive source of mesenchymal stem cells (MSCs) in the periodontium and are capable of regenerating cementum/PDL-like structures [4]. However, our previous studies revealed that PDLSCs obtained from periodontal tissues of periodontitis patients (PPDLSCs) are characterized by impaired function that leads to aberrant proliferative and osteogenic properties [5, 6].

Mechanical stimuli are another critical factor affecting tissue homeostasis and function. During orthodontic tooth movement, alveolar bone remodeling is triggered via initiation of a series of signaling cascades in the periodontal ligament (PDL) and surrounding tissues [7]. Nonetheless, inappropriate loading can cause homeostasis disruption between osteogenesis and bone resorption [8]. Our previous studies have also demonstrated that the reactions of PPDLSCs and HPDLSCs to static mechanical strain (SMS) differ due to the effect of the inflammatory microenvironment. Specifically, PPDLSCs display a sensitive pattern of both decreased proliferation and osteogenesis and an active inflammatory response to SMS at 12% elongation, while PDLSCs obtained from healthy periodontal tissues (HPDLSCs) exhibit a notable promotion of multidirectional capacities [6].

Long noncoding RNAs (lncRNAs) are an important type of molecule longer than 200 nucleotides that are transcribed by RNA polymerase II and contain a 5′ cap and 3′ adenylation [9]. Studies have identified that altered lncRNA levels are of functional importance in the pathogenesis and development of various diseases, including skeletal and dental diseases [10, 11]. In addition, lncRNA remodeling has been demonstrated to affect the progression of periodontitis [12]. For example, our group previously reported that the expression of lncRNA-POIR was reduced in PPDLSCs and that this was accompanied by decreased osteogenic capacity; moreover, overexpression of lncRNA-POIR promoted bone formation by competing with miR-182 [13].

Although many lncRNAs have been identified to be associated with inflammation-induced functional changes, the regulatory effects of lncRNAs on PPDLSCs in response to mechanical forces and the underlying mechanisms remain unclear [12, 14]. Therefore, this study was aimed at determining the SMS-induced lncRNA profiles of HPDLSCs and PPDLSCs and at exploring potential lncRNAs involved in the process of mechanotransduction in an inflammatory microenvironment.

## 2. Materials and Methods

### 2.1. Cell Culture

Primary PPDLSCs were obtained from premolar and/or third molar extractions of 8 donors (38.9 ± 7.9 years old) for therapeutic reasons who were diagnosed with chronic periodontitis. Primary HPDLSCs were isolated from 10 orthodontic patients (37.9 ± 7.2 years old) who underwent routine premolar and/or third molar extractions. All samples were collected at the Department of Orthodontics, School of Stomatology, the Fourth Military Medical University. Periodontitis patients were collected according to the following criteria made by the same periodontal specialist: bleeding on probe; periodontal pocket < 6 mm with 3-4 mm attachment loss; and/or alveolar bone absorption up to 1/3-1/2 root length horizontally on X-ray images. None of these subjects were selected with any clinical evidence of systemic disease or an acute infection in the past 6 months, and no one had a smoking history, drug utilization, or ever received maxillofacial radiotherapy and chemotherapy [6, 15]. All subjects provided written informed consent in accordance with the Declaration of Helsinki, and the study reached a consensus with the Ethics Committee of the Fourth Military Medical University (Approval Number: 2017(026)). The primary cells were cultured in *α*-MEM (Gibco BRL, Gaithersburg, MD, USA) with 10% fetal bovine serum (FBS) (Invitrogen, Carlsbad, CA, USA) in a humidified environment at 37°C with 5% CO_2_ [14]. Cell colonies were established by the limiting dilution technique [16].

### 2.2. SMS Loading

All cells were seeded into collagen I-coated 6-well BioFlex plates (Flexcell International, Burlington, NC, USA), and cells were serum starved for 24 h after achieving 95% confluence. Experimental cells were subjected to SMS for 12 h utilizing a Flexcell Tension Plus system (FX-4000T, Flexcell International) with 12% elongation at 0.1 Hz [6]. Static groups were cultured under the same conditions without SMS exposure.

### 2.3. RNA Extraction

Total RNA was extracted using TRIzol reagent (Invitrogen, Carlsbad, CA, USA) and quantified using a NanoDrop ND-1000 (Thermo Fisher Scientific, Boston, MA, USA). RNA integrity was evaluated by standard denaturing agarose gel electrophoresis.

### 2.4. Microarray Analysis

Sample labeling and array hybridization were conducted using the Agilent One-Color Microarray-Based Gene Expression Analysis protocol (Agilent Technologies, Santa Clara, CA, USA) [13]. In brief, mRNA was purified from total RNA after removing rRNA (mRNA-ONLY Eukaryotic mRNA Isolation Kit; Epicenter, San Diego, CA, USA). Each sample was amplified and transcribed into fluorescent cRNA along the entire length of the transcripts without 3′ bias via a random priming method (Arraystar Flash RNA Labeling Kit; Arraystar, Rockville, MD, USA). The labeled cRNAs were purified using an RNeasy Mini Kit (Qiagen, Hilden, Germany). The concentration and specific activity of the labeled cRNAs (pmol Cy3/*μ*g cRNA) were assessed using a NanoDrop ND-1000. One microgram of each labeled cRNA was fragmented by adding 5 *μ*l of 10× blocking agent and 1 *μ*l of 25× fragmentation buffer; after heating at 60°C for 30 min, 25 *μ*l of 2× GE hybridization buffer was added to dilute the labeled cRNA. Approximately 50 *μ*l of hybridization solution was dispensed into the gasket slide and assembled onto the lncRNA expression microarray slide. The slides were incubated for 17 at 65°C in an Agilent hybridization oven (Agilent, Santa Clara, CA, USA). The hybridized arrays were washed, fixed, and scanned using an Agilent DNA Microarray Scanner (part number G2505C). Arraystar Human LncRNA Microarray V3.0 (Arraystar, Kangchen, Shanghai, China) was designed for the global profiling of human lncRNAs and mRNAs. Approximately 30,586 lncRNAs and 26,109 coding transcripts can be detected by collecting data sources from GENCODE, UCSC, Ensembl, RefSeq, and other related sources.

### 2.5. Real-Time qPCR Confirmation

Total RNA was assembled and reverse-converted to cDNA using a SuperScript First-Strand Synthesis Kit (Invitrogen). An Applied Biosystems ViiA 7 Real-Time PCR System was used for qPCR. The reaction system included incubation for 10 min at 95°C, followed by 40 cycles at 95°C for 10 s and 60°C for 1 min. Relative expression levels of transcripts were calculated by using the 2^−*ΔΔ*CT^ method and normalized to GAPDH [17]. All experiments were carried out in triplicate. The specific primers (Genscript, China) used are shown in Table 1.

### 2.6. Bioinformatic Analysis of Differentially Expressed (DE) mRNAs

Gene Ontology (GO) analysis was employed to map DEmRNAs to GO terms annotated by molecular function, biological process, and cellular components (http://www.geneontology.org). Significant pathways of the DE genes were determined using Kyoto Encyclopedia of Genes and Genomes (KEGG) (http://www.genome.jp/kegg/), as previously described [18, 19].

### 2.7. Coexpression Network Construction (CNC)

CNC was conducted based on the top 10 DElncRNAs between strained HPDLSCs and PPDLSCs with coexpressed DEmRNAs [20]. Pearson's correlation coefficients (PPCs) no less than 0.99 were used to identify coding genes. CNCs were accomplished using Cytoscape software version 3.0.1 (The Cytoscape Consortium, San Diego, CA, USA).

### 2.8. Lentivirus Transfection

The design and construction of lentiviruses were performed by GeneChem (GeneChem, Shanghai, China). The lentivirus Ubi-MCS-SV40-EGFP-IRES-puromycin was used for lncRNA-XIST overexpression, and hU6-MCS-CBh-gcGFP-IRES-puromycin was used for lncRNA-XIST interference. The sequences of primers for amplifying lncRNA-XIST were F: 5′-ACAAGCAGTGCAGAGAGCT-3′ and R: 5′-AGAGTGCCAGGCATGTTGA-3′. The sequences of lncRNA-XIST interference targets were as follows: shlncRNA-XIST (79428-1): 5′-GCCATCATTAGCCACTGCACT-3′; shlncRNA-XIST (79428-2): 5′-GGTCAGGAGGTTCTGTCAAGA-3′; and shlncRNA-XIST (79428-3): 5′-GGTCCCAGATAGGAAGATAAA-3′. HPDLSCs and PPDLSCs were cultured in six-well plates. When cells reached approximately 30% confluence, they were transfected with lentiviruses at a multiplicity of transfection (MOI) of 9 for 24 h and then cultured with common medium (*α*-MEM with 10% FBS).

### 2.9. Osteogenic Differentiation Assays

For osteogenesis assays, HPDLSCs and PPDLSCs were exposed to SMS with 12% elongation at 0.1 Hz for 12 h after reaching 80% confluence. Then, HPDLSCs and PPDLSCs were cultured in osteogenic medium for 21 days. Mineralized nodules were stained with alizarin red S (pH 4.2) (Kermel, Tianjin, China) for 15 min at room temperature at day 21, and calcium levels were measured quantitatively using a calcium colorimetric assay kit (BioVision, San Francisco, CA, USA).

### 2.10. Statistical Analysis

All experiments were performed in triplicate, and the data are presented as the means ± standard deviation (S.D.). Statistical analyses with one-way ANOVA and Student's *t*-test were performed using SPSS 16.0 software (SPSS, San Rafael, CA, USA). Correlated terms were performed with the PCCs, and the significance threshold was fold change (FC) ≥ 2.0 and PCCs ≥ 0.9 and/or *P* < 0.05.

## 3. Results

### 3.1. Expression Profiles of DElncRNAs and DEmRNAs with SMS

**Figure 1:**
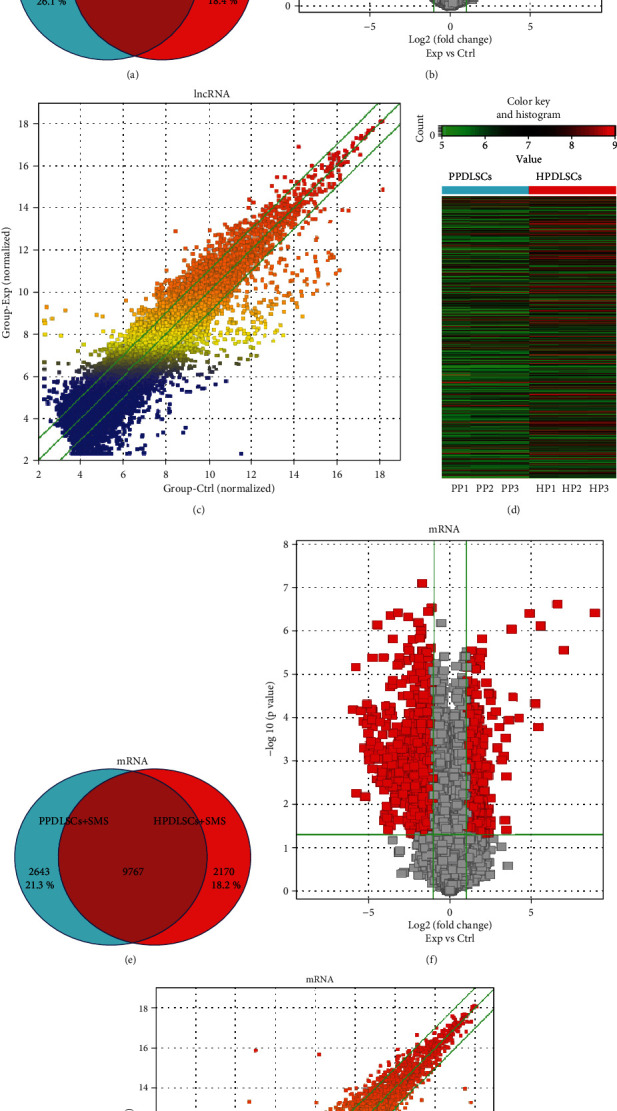
DElncRNAs and DEmRNAs between HPDLSCs and PPDLSCs after SMS exposure. Venn diagrams of DElncRNAs (a) and DEmRNAs (e). Volcano plot of expression profiles of lncRNAs (b) and mRNAs (f). Scatter plot of expression variations of lncRNAs (c) and mRNAs (g). Dots above the top and below the bottom green lines represent FC > 4.0. (d) Hierarchical clustering of DElncRNAs. Red represents relatively high expression, and green represents relatively low expression.

According to the principles of FC ≥ 2.0 and PPCs ≥ 0.9, we screened 8,847 and 9,772 DElncRNAs in strained HPDLSCs and PPDLSCs relative to static controls, respectively (Figure 1(a)). In addition, 1,624 DElncRNAs were only expressed in strained HPDLSCs, and 2,549 were only expressed in strained PPDLSCs. DElncRNAs with FC > 20.0 in each group are provided in Tables 2 and 3. Of those, ENST00000411904 was the most upregulated lncRNA in strained HPDLSCs; the most upregulated and downregulated lncRNAs in strained PPDLSCs were lncRNA-XIST and ENST00000517505, respectively. Volcano and scatter plots as well as hierarchical clustering were examined to assess the lncRNA expression differences between HPDLSCs and PPDLSCs exposed to the strain (Figures 1(b)–1(d)).

Thousands of DEmRNAs were identified (Figure 1(e)). In total, 11,937 and 12,410 DEmRNAs were significantly altered in strained HPDLSCs and PPDLSCs, respectively. In particular, 2,170 specific DEmRNAs were detected in strained HPDLSCs and 2,643 in strained PPDLSCs. The most upregulated and downregulated mRNAs in strained HPDLSCs were ASHGA5P006667 and ASHGA5P003418, and the most upregulated and downregulated mRNAs in strained PPDLSCs were ASHGA5P009176 and ASHGA5P052412 (KIF20A) (Tables 4 and 5). The volcano and scatter plots depicted in Figures 1(f) and 1(g) demonstrate the variation in lncRNA expression between strain-induced HPDLSCs and PPDLSCs.

### 3.2. Confirmation of DElncRNAs Using Real-Time qPCR

**Figure 2:**
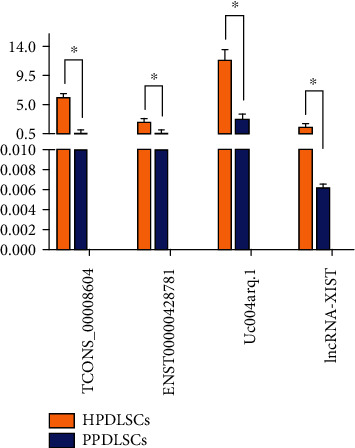
Real-time qPCR conformation of DE lncRNAs between strained HPDLSCs and PPDLSCs. All experiments were performed in triplicate, and the data are presented as the mean ± S.D.^∗^*P* < 0.05.

To validate the microarray results, we randomly selected four lncRNAs (TCONS_00008604, ENST00000428781, uc004arq.1, and XIST) from the top 10 DElncRNAs between strained HPDLSCs and PPDLSCs and evaluated their expression by qPCR assay (Table 6). All lncRNAs were downregulated in strained PPDLSCs compared to HPDLSCs, which was consistent with the microarray analysis results (Figure 2).

### 3.3. Preliminary Analysis of DEmRNAs with SMS

**Figure 3:**
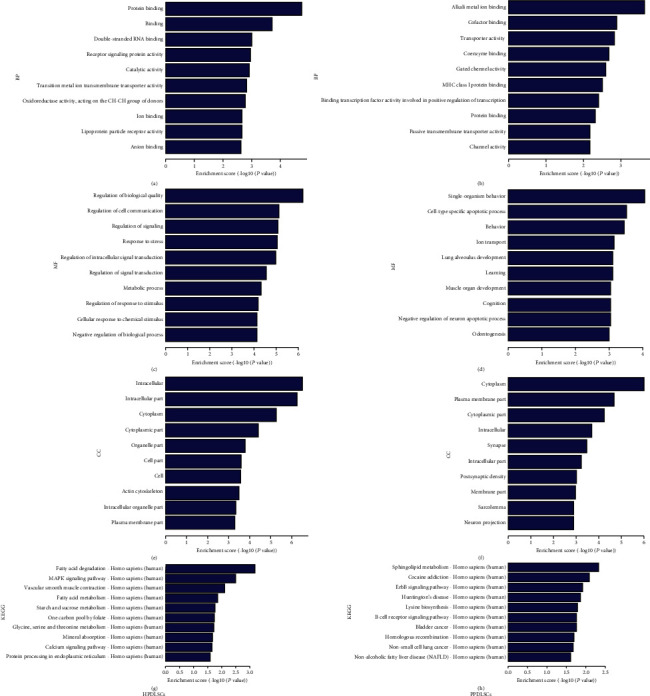
Top 10 functional analyses of DEmRNAs in strained HPDLSCs and PPDLSCs compared to static controls. Biological processes of DEmRNAs in strained HPDLSCs (a) and PPDLSCs (b). Molecular functions of DEmRNAs in strained HPDLSCs (c) and PPDLSCs (d). Cellular components of DEmRNAs in strained HPDLSCs (e) and PPDLSCs (f). KEGG pathways of DEmRNAs in strained HPDLSCs (g) and PPDLSCs (f).

To further explore the putative functions of lncRNAs, bioinformatic analysis was applied based on GO and KEGG pathway analyses. According to the results, DEmRNAs in strained HPDLSCs were mainly enriched in the regulation of stress response, signal transduction, and response to stimulus (Figures 3(a), 3(c), and 3(e)). In contrast, pathological processes such as cell-type apoptotic processes and neuronal apoptotic processes were enriched in strained PPDLSCs (Figures 3(b), 3(d), and 3(f)). Enrichment scores revealed prominent assignments for cellular function and metabolism, such as fatty acid degradation and metabolism, in strained HPDLSCs (Figure 3(g)). In strained PPDLSCs, pathological states were largely notable, including Huntington's disease, bladder cancer, and non-small-cell lung cancer (Figure 3(h)).

### 3.4. Constructions of the CNC Network

**Figure 4:**
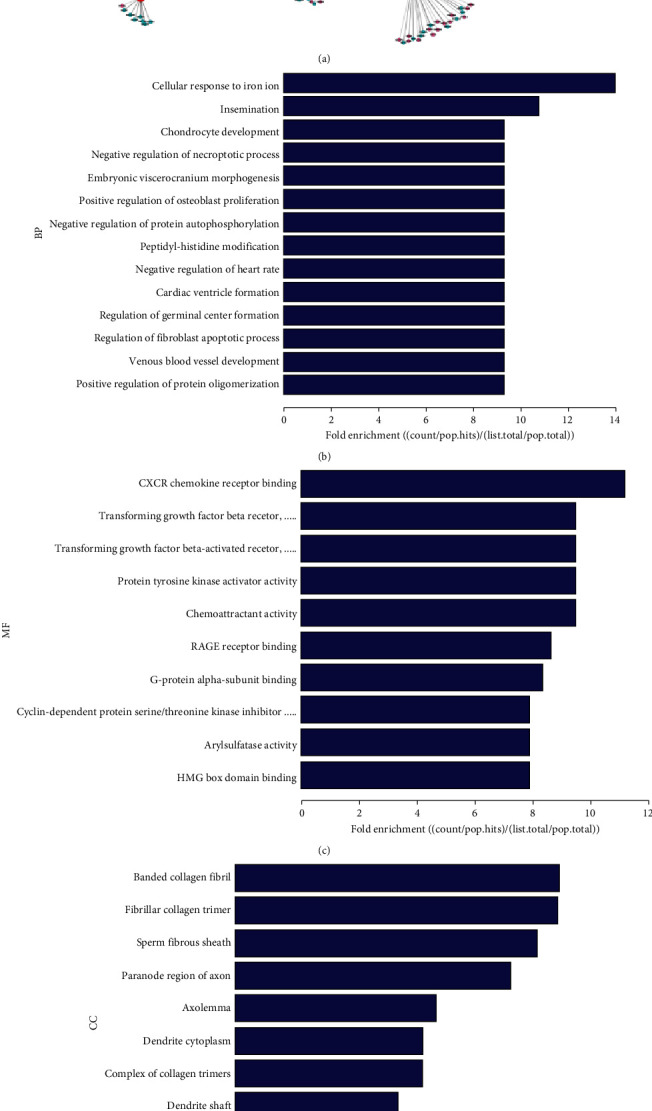
Functional analysis of the top 10 DElncRNAs associated with DEmRNAs between strained HPDLSCs and PPDLSCs. (a) lncRNA-mRNA interaction network of the top 10 DElncRNAs related to mRNAs. The red and blue spots represent upregulated lncRNAs and downregulated lncRNAs, respectively. In addition, the green and pink colors represent upregulated coding genes and downregulated coding genes, respectively. Top 10 biological processes (b), molecular functions (c), and cellular components (d) of the differences in coexpressed lncRNAs. (e) KEGG pathways of the differences in coexpressed lncRNAs.

By combining the top 10 DElncRNAs with coexpressed DEmRNAs, an integrated coexpression network containing 1,250 lncRNA-mRNA interactions was established (Figure 4(a)). Notably, RP11-597D13.9 (ENST00000505532) was associated with the maximum number of DEmRNAs, up to 160. XIST, the most downregulated lncRNA in strained PPDLSCs, was coexpressed with 47 DEmRNAs (Table 6). In addition, GO annotations and KEGG analyses showed that the DElncRNAs in the key module are related to chondrocyte development, fibroblast apoptotic process regulation, and cell adhesion as well as leukocyte transendothelial migration, which likely participate in tissue regeneration. Taken together, dysregulated lncRNAs are involved in the pathological modification of gene expression in PPDLSCs under mechanical conditions (Figures 4(b)–4(e)).

### 3.5. Functional Investigation of DElncRNAs during Osteogenic Differentiation

**Figure 5:**
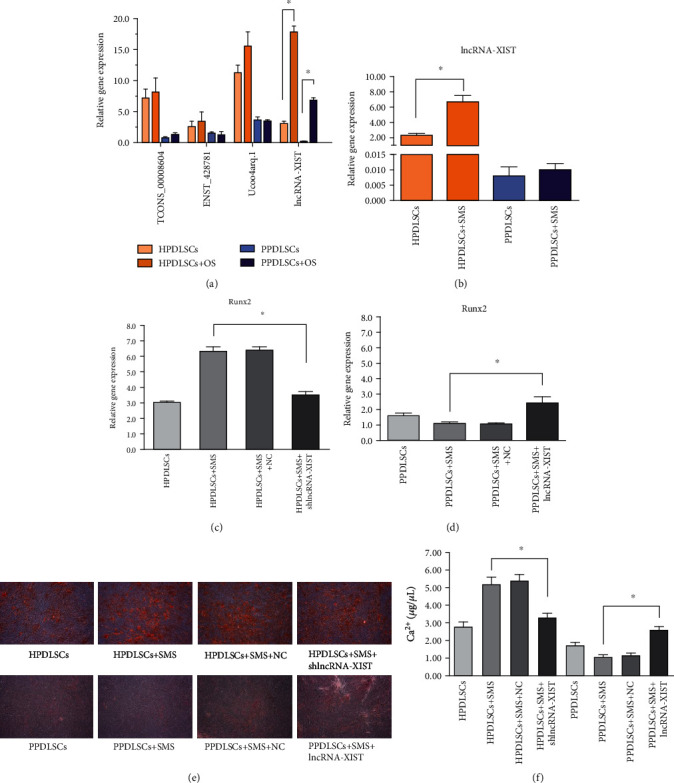
The expression levels of DElncRNAs in HPDLSCs and PPDLSCs during osteogenic differentiation: (a) the expression levels of DElncRNAs in HPDLSCs and PPDLSCs after osteogenic differentiation for 7 days; (b) the expression levels of lncRNA-XIST in strained HPDLSCs and PPDLSCs; (c) detection of the osteogenic gene Runx2 in strained HPDLSCs infected with shlncRNA-XIST; (d) detection of the osteogenic gene Runx2 in strained PPDLSCs infected with lncRNA-XIST; (e) alizarin red staining for HPDLSCs and PPDLSCs; (f) calcium quantification for HPDLSCs and PPDLSCs. All experiments were performed in triplicate, and the data are presented as the mean ± S.D. Scale bar = 100 *μ*m.

We randomly evaluated the expression of four lncRNAs (TCONS_00008604, ENST00000428781, uc004arq.1, and XIST) from the top 10 DElncRNAs between strained HPDLSCs and PPDLSCs after osteogenic induction for 7 days and observed that the expression level of lncRNA-XIST significantly increased in HPDLSCs at day 7 after osteogenic differentiation. Although osteogenic induction upregulated the level of lncRNA-XIST in PPDLSCs, it was still lower than that in HPDLSCs (*P* < 0.05, Figure 5(a)). We also found that lncRNA-XIST was significantly increased in HPDLSCs after 12 h of SMS elongation (*P* < 0.05); however, strain-induced lncRNA-XIST expression was not obviously increased in PPDLSCs (Figure 5(b)). We also examined the relationship between lncRNA-XIST and the osteogenic gene Runx2 after 12% SMS loading by lentivirus transfection. We found that Runx2 expression in strained HPDLSCs infected with shlncRNA-XIST was decreased almost 2 times compared with strained HPDLSCs in the negative control group (NC) (*P* < 0.05, Figure 5(c)). In contrast, Runx2 expression in strained PPDLSCs increased after lncRNA-XIST overexpression (*P* < 0.05, Figure 5(d)). Similarly, alizarin red staining and calcium quantification also sustained that shlncRNA-XIST inhibited SMS-induced osteogenic differentiation in HPDLSCs and that overexpression of lncRNA-XIST rescued the osteogenic ability of PPDLSCs (*P* < 0.05, Figures 5(e) and 5(f)).

## 4. Discussion

Many lncRNAs play critical roles in multiple pathological processes of periodontitis, such as proliferation, differentiation, cell migration, and immune regulation [21, 22]. Excessive mechanical stimuli can cause irreversible damage to PDLSCs, especially to those in an inflammatory state [23]. However, studies on the expression of lncRNAs involved in strain-induced PDLSCs and their potential effects on cellular functions are limited. In this study, we identified thousands of DElncRNAs and DEmRNAs in HPDLSCs and PPDLSCs after SMS application, and various lncRNAs and mRNAs were found to be solely expressed in strained HPDLSCs or PPDLSCs, indicating that different mechanisms may be involved in the mechanotransductive responses of PDLSCs derived from different contexts.

Using microarray analysis, we observed that DElncRNAs in strained HPDLSCs were mainly enriched in mechanoconductive processes; pathological pathways such as cell-type apoptotic process and regulation of neuron apoptotic process were associated with dysregulated lncRNAs in strained PPDLSCs. In addition, based on KEGG pathway analysis, the DElncRNAs are largely related to pathological conditions such as Huntington's disease [24], bladder cancer [25], and non-small-cell lung cancer [26]. Therefore, cell functions regulated by lncRNAs have a potential role in PDLSCs, and aberrant lncRNA transcripts are associated with periodontitis progression [27].

Cytoskeletal dynamics and integrity are of vital importance for cell differentiation commitment, by which the bone loss occurring in periodontitis can be alleviated [28]. By altering the expression of eukaryotic cytoskeleton proteins, which play important roles in cancer progression and cytoskeleton modulation, KIF20A is sensitive to alterations along with mechanical loadings [29]. In this study, KIF20A was most downregulated in strained PPDLSCs, suggesting that dysregulated mRNAs possibly modulate expression through interactions with intracellular cytoskeleton mechanisms. Therefore, further functional validation of these dysregulated transcripts in periodontitis is warranted.

Furthermore, to identify potential lncRNAs associated with strained PDLSCs, we integrated the coexpression networks of lncRNAs and mRNAs. A total of 1,250 pairs based on the top 10 dysregulated lncRNAs between strained PPDLSCs and HPDLSCs were established. Of those, RP11-597D13.9, an antisense lncRNA, correlated with up to 160 mRNAs, and it may affect a nearby coding gene: FAM198B [30]. FAM198B has been implicated as a tumor inhibitor, attenuating lung cancer cell invasion and thus improving the overall survival of patients with lung adenocarcinoma [31]. In our study, RP11-597D13.9 displayed an inverse trend of downregulation in strained PPDLSCs, indicating a possible pathological state for these cells. Moreover, lncRNA XIST has long been recognized as an oncogenic gene and is preferentially expressed in cancers [32, 33]. LPS-induced inflammation can increase the levels of XIST expression, which in turn suppresses acute inflammation via MAPK signaling [34]. Contrary to these results, there was a significant decrease in XIST in strained PPDLSCs together with CH1CI (Brx), one of the major downstream target genes for XIST [35]. In our study, we first found that lncRNA-XIST expression decreased in PDLSCs derived from an inflammatory microenvironment. Additionally, although SMS elongation significantly decreased the expression of lncRNA-XIST in PPDLSCs compared with HPDLSCs, the upregulation of lncRNA-XIST in strained PPDLSCs increased the process of osteogenic differentiation, indicating that lncRNA-XIST may be one of the nonnegligible reasons for the impaired osteogenesis in strained PPDLSCs.

## 5. Conclusions

In summary, differentially expressed lncRNA profiles between HPDLSCs and PDLSCs under mechanical exposure were first identified in this study, and many were specifically expressed. By functional analysis, we confirmed that DE transcripts in PPDLSCs participate in many pathological processes and might be involved in regulating periodontitis progression under tension. In our study, we found that the expression of lncRNA-XIST obviously decreased in PPDLSCs, and the osteogenic ability of PPDLSCs under tension loading was significantly upregulated after upregulation of lncRNA-XIST by lentivirus. These results hint us that lncRNAs could regulate the osteogenic ability of PDLSCs under tension loading. Although some lncRNAs are predicted, comprehensive analyses are still needed to elucidate the details of the relevant molecular mechanisms.

## Figures and Tables

**Table 1:** Primers designed for real-time qPCR validation of candidate lncRNAs.

Gene symbol	Sense primer
GAPDH (HUMAN)	F: 5′GGGAAACTGTGGCGTGAT3′ R: 5′GAGTGGGTGTCGCTGTTGA3′
TCONS_00008604	F: 5′GTTGGGCAGTAAGCCTCACA3′ R: 5′TGGGGTAGGTAATGGAAAAAG3′
ENST00000428781	F: 5′AGGGGGTAAAAGAAAATGGTG3′ R: 5′CAGGCTCGCATTCAGACAT3′
uc004arq.1	F: 5′ACCCCTACAGACCATAACAAAG3′ R: 5′AGCCGACTACAGCCACCACT3′
XIST	F: 5′GCTGAATGAATGTGTCTTACCC3′ R: 5′GAGGCAAAGGCACACACGAA3′
Runx2	F: 5′CCCGTGGCCTTCAAGGT-3′
	R: 5′CGTTACCCGCCATGACAGTA-3′

**Table 2:** DElncRNAs with FC > 20.0 in strained HPDLSCs compared to static controls.

Sequence name	Source	Fold change	Regulation	*P* value (×10^−4^)
NR_038400	RefSeq	35.06	Up	0.000
ENST00000423442	GENCODE	35.06	Up	0.024
ENST00000409139	GENCODE	33.15	Up	0.229
ENST00000432511	GENCODE	31.93	Up	0.039
ENST00000433431	Pseudogene	31.57	Up	0.169
ENST00000453278	GENCODE	30.70	Up	0.358
uc003 mjk.3	UCSC_knowngene	29.42	Up	24.755
ENST00000447956	GENCODE	28.82	Up	0.047
ENST00000505532	GENCODE	28.06	Up	0.235
TCONS_00024645	LincRNAs identified by Cabili et al.	27.71	Up	0.451
TCONS_00010599	LincRNAs identified by Cabili et al.	24.92	Up	0.659
DB299803	LincRNAs identified by Khalil et al.	23.18	Up	0.115
TCONS_00008978	LincRNAs identified by Cabili et al.	21.99	Up	0.784
ENST00000506014	GENCODE	20.49	Up	1.714

**Table 3:** DElncRNAs with FC > 20.0 in strained PPDLSCs compared to static controls.

Sequence name	Source	Fold change	Regulation	*P* value (×10^−4^)
XIST	GENCODE	53.85	Down	1.089
ENST00000517505	GENCODE	50.01	Up	107.825
ENST00000583761	GENCODE	40.94	Up	5.450
TCONS_00024405	LincRNAs identified by Cabili et al.	37.30	Down	0.059
ENST00000523905	GENCODE	36.91	Up	20.468
TCONS_00008604	LincRNAs identified by Cabili et al.	34.05	Down	0.795
AA324424	LincRNAs identified by Khalil et al.	33.45	Up	0.048
ENST00000545920	GENCODE	33.25	Down	3.759
ENST00000486545	GENCODE	27.35	Down	0.003
TCONS_00019524	LincRNAs identified by Cabili et al.	25.44	Up	4.413
ENST00000428781	GENCODE	24.73	Down	0.259
uc004arq.1	UCSC_knowngene	22.42	Down	0.059
TCONS_00014003	LincRNAs identified by Cabili et al.	21.10	Up	20.461

**Table 4:** DEmRNAs with FC > 20.0 in strained HPDLSCs compared to static controls.

Sequence name	Source	Fold change	Regulation	*P* value (×10^−5^)
ASHGA5P006667	RefSeq	413.31	Up	4.315
ASHGA5P008770	RefSeq	147.59	Up	0.0003
ASHGA5P021973	RefSeq	125.83	Up	150.671
ASHGA5P013772	GENCODE	70.35	Up	1512.671
ASHGA5P003418	RefSeq	63.94	Down	74.253
ASHGA5P011737	RefSeq	42.60	Up	494.192
ASHGA5P007165	GENCODE	41.99	Up	302.842
ASHGA5P037277	RefSeq	41.81	Up	1.06.612
ASHGA5P005733	GENCODE	38.72	Up	46.774
ASHGA5P002830	GENCODE	37.77	Up	15.020
ASHGA5P002962	RefSeq	36.22	Up	5.402
ASHGA5P013771	RefSeq	33.74	Up	46.840
ASHGA5P042689	RefSeq	32.07	Up	92.410
ASHGA5P002150	RefSeq	31.42	Up	0.128
ASHGA5P007745	RefSeq	30.45	Up	6.915
ASHGA5P003294	GENCODE	28.31	Up	0.254
ASHGA5P017399	RefSeq	28.24	Up	7.840
ASHGA5P008733	GENCODE	25.41	Up	64.712
ASHGA5P005403	RefSeq	23.76	Up	1.361
ASHGA5P054134	RefSeq	23.62	Up	245.163
ASHGA5P001729	RefSeq	23.04	Up	14.825
ASHGA5P051262	RefSeq	22.99	Up	858.448
ASHGA5P045542	RefSeq	22.91	Up	200.313
ASHGA5P050260	RefSeq	22.54	Up	0.001
ASHGA5P004313	RefSeq	21.00	Up	4.695
ASHGA5P001728	RefSeq	20.64	Up	84.501

**Table 5:** DEmRNAs with FC > 20.0 in strained PPDLSCs compared to static controls.

Sequence name	Source	Fold change	Regulation	*P* value (×10^−5^)
ASHGA5P009176	RefSeq	111.34	Up	179.785
ASHGA5P013422	RefSeq	59.61	Up	6.424
ASHGA5P010424	GENCODE	56.12	Up	0.032
ASHGA5P012978	RefSeq	42.19	Up	104.171
ASHGA5P052412	RefSeq	42.10	Down	0.001
ASHGA5P003780	RefSeq	34.88	Up	2427.963
ASHGA5P005903	RefSeq	31.08	Down	0.919
ASHGA5P017401	RefSeq	27.31	Down	0.304
ASHGA5P001619	RefSeq	25.51	Up	784.373
ASHGA5P004428	RefSeq	24.18	Down	26.041
ASHGA5P004064	RefSeq	21.49	Down	4.546
ASHGA5P034395	GENCODE	20.98	Up	55.833

**Table 6:** Top 10 DElncRNAs between strained HPDLSCs and PPDLSCs with coexpressed mRNAs.

Sequence name	Total mRNA
ENST00000505532	160
ENST00000532307	156
ENST00000428781	126
uc021qut.1	130
TCONS_00008604	93
uc004arq.1	51
ENST00000423727	89
TCONS_00013636	65
XIST	47
ENST00000340196	12

## Data Availability

The data used to support the findings of this study are available from the corresponding author upon request.
